# Activation of AKR1C1/ERβ induces apoptosis by downregulation of c-FLIP in prostate cancer cells: A prospective therapeutic opportunity

**DOI:** 10.18632/oncotarget.3417

**Published:** 2015-03-24

**Authors:** Huiyoung Yun, Jianping Xie, Aria F. Olumi, Rita Ghosh, Addanki P. Kumar

**Affiliations:** ^1^ Department of Urology, The University of Texas Health Science Center, San Antonio, TX, USA; ^2^ Department of Pharmacology, The University of Texas Health Science Center, San Antonio, TX, USA; ^3^ Department of Urology, Massachusetts General Hospital Harvard Medical School, Boston, MA, USA; ^4^ Department of Molecular Medicine, The University of Texas Health Science Center, San Antonio, TX, USA; ^5^ Cancer Therapy and Research Center, The University of Texas Health Science Center, San Antonio, TX, USA; ^6^ South Texas Veterans Health Care System, San Antonio, TX, USA; ^7^ Department of Urology, Shanxi Dayi Hospital, Shanxi Academy of Medical Science, Taiyuan, P.R., China

**Keywords:** androgen receptor, estrogen receptor beta, c-FLIP, 2-methoxyestradiol, transcription factor

## Abstract

We provide first-time evidence for ERβ-mediated transcriptional upregulation of c-FLIP as an underlying mechanism in the development of castrate-resistant cancer. While androgens inhibit apoptosis partly through transcriptional upregulation of the anti-apoptotic protein, c-FLIP in androgen-responsive cells, they downregulate c-FLIP in androgen-independent cells. We found that although Sp1 and p65 trans-activate c-FLIP, the combination of Sp1 and p65 has differential effects in a cellular context-dependent manner. We show that activation of the androgen metabolism enzyme, aldo-keto reductase, AKR1C1, relieves androgen independence through activation of 3β-Adiol-mediated upregulation of ERβ. ERβ competes with Sp1 and Sp3 to transcriptionally downregulate c-FLIP in the absence of consensus estrogen-response element in androgen-independent cells. Forced expression of AR in androgen-independent cells show that ERβ-mediated growth inhibition occurs under conditions of androgen independence. Reactivation of ERβ with the estrogenic metabolite, 2-methoxyestradiol, decreased enrichment ratio of Sp1/Sp3 at the c-FLIP promoter with concomitant effects on cell growth and death. Expression of Sp1 and c-FLIP are elevated while AKR1C1, ERβ and Sp3 are significantly low in human prostate tumor samples. ERβ is epigenetically silenced in prostate cancer patients, therefore our results suggest that combination of ERβ agonists with ADT would benefit men stratified on the basis of high estrogen levels.

## INTRODUCTION

The binding of androgens to the androgen receptor (AR) plays an important role not only in the growth and development of normal prostate, but also in prostatic diseases including prostate cancer (PCA). Accordingly, androgen deprivation therapy (ADT) is the mainstay therapeutic approach for locally advanced PCA [1]. Although initially effective, the outcome is transient, invariably resulting in progression to aggressive metastatic castrate-resistant form (CRPC) with no effective curative options [2]. ADT functions by causing apoptotic cell death of cancer cells. However, advanced prostate cancer cells develop resistance to ADT-induced apoptosis leading to development of aggressive castrate-resistant status [3]. Additionally, though PCA is an androgen-dependent disease, levels of androgens decrease while estrogens increase with age [4]. Accordingly, it was suggested that rather than androgens *per se*, the ratio of androgen to estrogens may be a potential reason for development of CRPCA [5]. This is corroborated by emerging evidence implicating a role of estrogen receptors (ERs) primarily α and β (ERα and β) in prostate cancer cells and human prostate tumors [6, 7]. Furthermore, ectopic expression of ERβ inhibits growth, migration, invasion and epithelial mesenchymal transition of prostate cancer cells and ERβ knockout mice develop hyperplasia and PIN lesions [7, 8]. However, the underlying mechanism of ERβ-mediated prostate pathogenesis including CRPCA is undefined. Studies to understand the molecular events associated with CRPCA identified an important role for the anti-apoptotic FLICE-inhibitory protein (c-FLIP) [11]. c-FLIP is aberrantly expressed in high-grade and castrate-resistant human prostate tumors. Furthermore, nude mice implanted with c-FLIP overexpressing LNCaP cells develop androgen independent prostate tumors suggesting androgen regulation of c-FLIP. Evidence from epidemiological and laboratory studies indicate lower PCA incidence in men consuming soy products [12]. Gut microbial digestion of these products generates estrogenic metabolites with anti-tumorigenic activity [12]. Along these lines, studies from number of laboratories including our own demonstrated anti-tumorigenic activity for a non-toxic endogenous estrogenic metabolite 2-methoxyestradiol (2-ME_2_) in multiple tumor types *in vitro* and *in vivo* [13–17]. In addition, downregulation of c-FLIP by 2-ME_2_ has been reported to inhibit tumor growth *in vitro* and *in vivo* [13–17]. Despite the circumstantial evidence, the underlying mechanism through which androgens and loss of ERβ influence c-FLIP deregulation during prostate carcinogenesis and whether 2-ME_2_-mediated inhibition of prostate tumor development involves ERβ/c-FLIP remains to be defined.

Here we investigated the functional interaction between androgen metabolism-mediated activation of ERβ as a possible underlying mechanism involved in deregulation of c-FLIP. We provide evidence that c-FLIP is negatively regulated by ERβ possibly through modulation of Sp1/Sp3 binding to its promoter. We also provide evidence that inhibition of Sp1 activation coupled with ERβ activation with 2-ME_2_ suppresses tumor cell growth and induces apoptosis. These findings identify ERβ as a negative modulator of c-FLIP and suggest strategies to target ERβ activation either directly or by enhancing androgen metabolism enzyme AKR1C1 along with AR inhibition as a novel approach for effective management of CRPCA.

## RESULTS

### Differential regulation of c-FLIP by Sp1 and NFκB in prostate cancer cells

**Figure 1:**
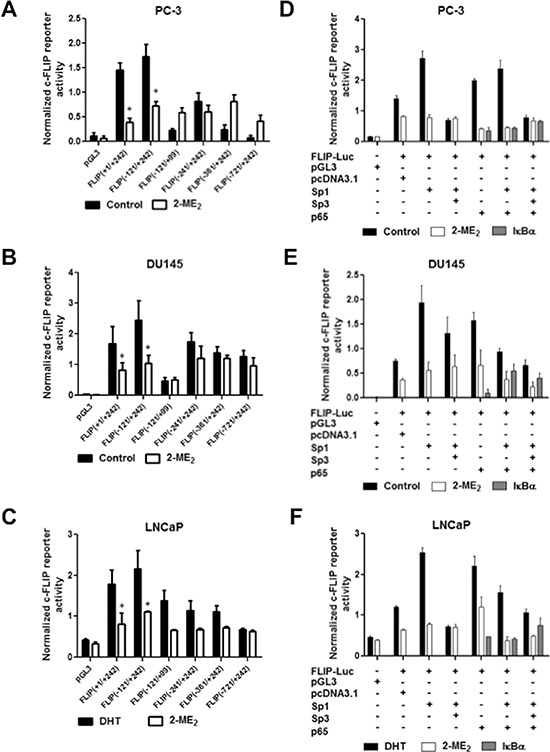
Identification of core c-FLIP promoter elements sufficient for constitutive and 2-ME2 response, and regulation of c-FLIP with multiple transcription factors including Sp1, Sp3 and NFκB **A–C.** Androgen independent PC-3, DU145, and androgen-responsive LNCaP cells were transfected with indicated deletion constructs of c-FLIP reporter plasmid (0.5 μg). Post-transfection, cells were treated with 2-ME_2_ (3 μM for LNCaP and PC-3, and 5 μM for DU145) for 24 h. Luciferase activity was measured. Cells transfected with pGL3 empty vector was used as negative control. Data presented is average ± S.E.M of three independent experiments conducted in triplicate. *p < 0.05. **D–F.** PC-3, DU145 and LNCaP cells were co-transfected with pGL3-FLIP (–121/+242)-Luc plasmid along with empty vector (pCMV3.1) or expression plasmids for (pCMV-Sp1, pCMV-Sp3, pCMV-p65 or IκBα mutant, a super repressor of NFκB (0.5 μg/well of each). Post-transfection, cells were treated with 2-ME_2_ for 24 h and luciferase activity was measured. Cells transfected with empty vector was used as negative control. Data presented is average ± S.E.M of three independent experiments conducted in triplicate.

Although published studies have demonstrated that 2-ME_2_ inhibits prostate cancer cell growth by suppressing transcriptional activation of c-FLIP, the molecular mechanism through which 2-ME_2_ suppresses c-FLIP activation is not defined [13]. Transient expression assays using exonuclease deletion constructs spanning the 5′-flanking region of c-FLIP promoter element identified –121/+242 sequence with maximal constitutive reporter activity in both androgen-responsive LNCaP and androgen-independent prostate cancer cells PC-3 and DU145 cells (Fig. 1A–1C). Interestingly, promoter activity increased significantly in response to androgens (5α-DHT stimulation) in LNCaP cells (Fig. 1C and data not shown). Constitutive c-FLIP promoter activity in PC-3 and DU145 cells or 5α-DHT-stimulated activity in LNCaP cells decreased following treatment with 2-ME_2_. Inclusion of sequence elements upstream of –121 not only decreased the basal promoter activity but also the 2-ME_2_ response. These data suggest that 2-ME_2_ response elements are located within sequence elements –121/+242 and that this sequence element was sufficient to maintain the c-FLIP core promoter activity.

Analysis of –121/+242 sequence identified putative binding sites for multiple transcription factors including AR, Sp1 and NFκB (Supplementary Fig. S1A). Therefore, we tested the impact of ectopic expression of Sp1, Sp3 and p65 (NFκB) on c-FLIP promoter activity using co-transfections. Ectopic expression of Sp1 or p65 transactivated c-FLIP in all three prostate cancer cell lines (Fig. 1D–1F). The observed p65-mediated transactivation was repressed in cells co-transfected with phosphorylation defective IκBα indicating the specificity of NFκB-mediated effects. Interestingly, we observed differential effects of co-transfection with Sp1 on p65 transactivation of c-FLIP promoter. Sp1 repressed p65 transactivation of c-FLIP in DU145 and LNCaP cells (Fig. 1E–1F) with no significant effect in PC-3 cells (Fig. 1D). Furthermore, ectopic expression of Sp3 or treatment with 2-ME_2_ inhibited both Sp1 and p65-mediated transactivation (Fig. 1D–1F). These data suggest (i) Sp1 as master regulator of c-FLIP; and (ii) either Sp1 or p65 can individually transactivate c-FLIP, and that the combination of Sp1 and p65 can have differential effects depending on the cell type.

### Identification and characterization of DNA-protein complexes binding to c-FLIP promoter

**Figure 2:**
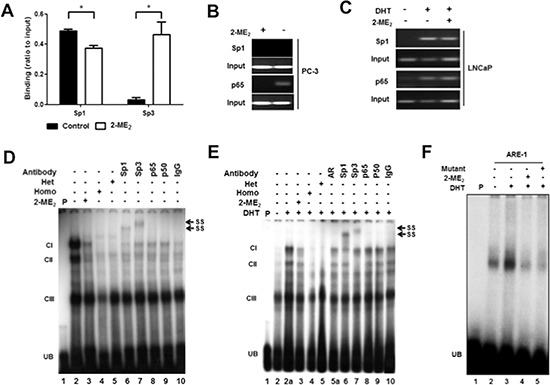
Sp1, Sp3 and NFκB bind to the endogenous c-FLIP promoter **A.** DU145 cells were untreated or treated with 2-ME_2_ (5 μM) for 24 h, and chromatin immunoprecipitation quantitative real-time PCR (ChIP-qPCR) was performed using anti-Sp1 or anti-Sp3 antibody. The amplification value from immunoprecipitated DNA was normalized to 10% input. Error bars indicate ± S.E.M. (*n* = 3). *p < 0.05. **B.** PC-3 cells were untreated or treated with 2-ME_2_ (5 μM) for 24 h. **C.** LNCaP cells were untreated or treated with 2-ME_2_ (3 μM) for 6 h in the presence or absence of DHT (1 nM). Gel-based ChIP-PCR was performed using anti-Sp1 or anti-p65 antibody. **D.** Binding of AR, Sp1, Sp3 and p65 to the c-FLIP promoter sequence elements containing Sp1, NFκB, or AR binding sites to nuclear extracts prepared from PC-3 cells using electrophoretic mobility shift assay (EMSA). PC-3 cells untreated or treated with 2-ME_2_ (3 μM) for 24 h. Radiolabeled c-FLIP probe was preincubated with 100-fold molar excess of unlabeled c-FLIP sequence (homologous) or Sp1 oligonucleotide with mutation (heterologous) for 5 min prior to incubation with nuclear extracts. Nuclear extracts pre-incubated with indicated antibodies for 30 minutes on ice were used in super-shift experiments. **E.** Nuclear extracts prepared from LNCaP cells untreated or pretreated with 2-ME_2_ (3 μM) for 6 h prior to stimulation with DHT (1 nM) for 1 h were used in EMSA. EMSA was carried out essentially as described above. **F.** Binding of nuclear extracts from untreated or 5α-DHT stimulated LNCaP cells to c-FLIP ARE-1 (+57/+71) as radiolabeled probe was shown. (CI-III: DNA-protein complexes, UB: unbound free probe, SS: super-shifted bands).

Chromatin immunoprecipitation assay (ChIP) was used to examine whether Sp1 regulates endogenous c-FLIP in the context of chromatin using overlapping binding sites for Sp1 and p65 (Supplementary Fig. S1B). Chromatin immunoprecipitated extracts from untreated DU145 but not PC-3 cells with anti-Sp1 antibody amplified c-FLIP sequence (Fig. 2A and 2B). It is noteworthy to mention here that Sp1 was recruited to the c-FLIP sequences upstream of –833 (data not shown). In contrast, in LNCaP cells significant enrichment of Sp1 was observed only under androgen-stimulated conditions (Fig. 2C). Treatment with 2-ME_2_ reduced Sp1 but enhanced Sp3 enrichment in DU145 cells with no significant effect in LNCaP cells. No significant change in binding to *β*-actin (negative control) was observed under these conditions. It should be noted that in transient expression assays Sp1 transactivates c-FLIP promoter in PC-3 cells. These surprising findings lead us to examine p65 binding (given its overlap with Sp1 site) to endogenous c-FLIP. Interestingly, chromatin immunoprecipitated extracts with anti-p65 specifically bound these sequences that was abolished with 2-ME_2_ in PC-3 cells (Fig. 2B). Taken together, these data suggest multiple transcription factors including Sp1, Sp3 and NFκB differentially regulate c-FLIP depending on the cell type and recruitment of both Sp1 and p65 to overlapping sites of c-FLIP promoter.

To examine if Sp1 and p65 regulate c-FLIP by binding to the same sequence elements, gel super-shift experiments were conducted using c-FLIP promoter sequence (+64/+89 containing AR, Sp1 and NFκB overlapping bindings sites; Supplementary Fig. S1C) as radiolabeled probe. Given our data suggesting differential regulation of c-FLIP between LNCaP and DU145 vs. PC-3, we performed gel shift experiments using LNCaP and PC-3 cells. Nuclear extracts from PC-3 or 5α-DHT-stimulated LNCaP cells formed three distinct DNA-protein complexes (Complex I, II and III; Fig. 2D and 2E). The observed complexes were either completely (CI and II) or partially (CIII) abolished in response to 2-ME_2_ treatment (lane 3). Competition experiments using c-FLIP oligo or mutant Sp1 oligonucleotide as homologous or heterologous competitors respectively abolished both CI and II but CIII was partially abolished (lanes 4 and 5). These data suggest the presence of Sp1, Sp-related and factors other than Sp1 in these complexes. Pre-incubation of nuclear extracts with antibodies against Sp1 and Sp3 showed super-shifted complexes (lanes 6 and 7; indicated as SS) or reduced complex formation with p65 and p50 (lanes 8 and 9) in both cell types. In addition, pre-incubation of nuclear extracts from LNCaP cells with AR antibody partially abolished the observed DNA-protein complex indicating the presence of AR in androgen-responsive cells (lane 5a).

Previous studies have demonstrated the presence of androgen response element (ARE)-1 within this sequence [20]. These data prompted us to perform gel shift experiments using c-FLIP sequence containing wild type ARE-1 (Supplementary Fig. S1D). 5α-DHT-stimulated nuclear extracts from LNCaP cells showed enhanced binding to this sequence that was reduced in response to 2-ME_2_ (lanes 3 and 4; Fig. 2F). Competition experiments using ARE1 with mutation in conserved binding site eliminated the observed DNA-protein complex indicating the presence of factors other than AR, including Sp1 and p65 (lane 5; Fig. 2F). Taken together, these data show the presence of AR, Sp1 and p65 in CI and Sp3 in CII and suggest that AR bound c-FLIP promoter sequence containing Sp1/NFκB sequences is upregulated in response to androgens in LNCaP cells.

### Androgen down regulate c-FLIP activation in androgen independent cells

**Figure 3:**
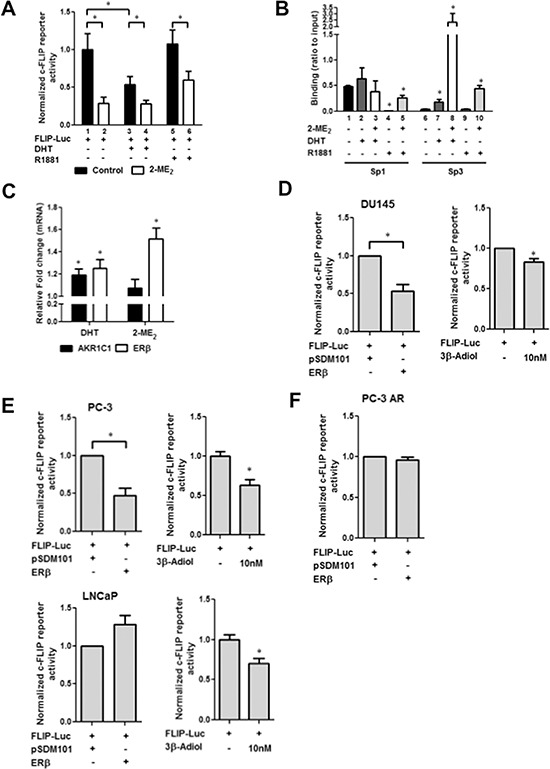
Androgen metabolism regulates c-FLIP transcriptional activity **A.** 5α-DHT but not R1881 inhibits c-FLIP promoter activity. DU145 cells were transfected with c-FLIP reporter plasmid (0.5 μg). Post-transfection, cells were serum-starved for additional 24 h, treated with 2-ME_2_ (5 μM) for 24 h and then stimulated with 5α-DHT or R1881 (1 nM) for 1 h. Luciferase activity was determined. Error bars represent ± S.E.M (*n* = 3). *p < 0.05 (Student's *t*-test). **B.** Binding of Sp1 and Sp3 to the endogenous c-FLIP promoter stimulated with DHT or R1881 in the presence or absence of 2-ME_2_. After stimulation with 5α-DHT or R1881 (1 nM) for 1 h and/or treatment with 2-ME_2_ for 24 h, ChIP-qPCR was performed using anti-Sp1 or anti-Sp3 antibody in DU145 cells. The amplification value from immunoprecipitated DNA was normalized to 10% input. Error bars represent ± S.E.M. (*n* = 3). *p < 0.05. **C.** AKR1C1 and ERβ mRNA expression in DU145 cells treated with 5α-DHT or 2-ME_2_. Total RNA was isolated from cells treated with 5α-DHT or R1881 (1 nM) for 1 h, or with 2-ME_2_ (5 μM) for 24 h. PCR reactions were conducted in triplicate, and relative mRNA expression was normalized to *β*-actin. Error bars represent ± S.D. (*n* = 3). *p < 0.05. **D–F.** ERβ regulates c-FLIP in prostate cancer cells. pGL3-FLIP (–121/+242)-Luc plasmid was co-transfected along with empty vector (pSDM101) or expression plasmids for ERβ (0.5 μg/well). Post-transfection, cells were treated with 2-ME_2_ for 24 h and luciferase activity was measured. In addition, cells were transfected with pGL3-FLIP (–121/+242)-Luc plasmid and treated with different concentration of 3β-Adiol. Panels D, E, and F represent DU145, PC-3 (Fig. 3E top panel), LNCaP (Fig. 3E bottom panel) and PC-3 AR (PC-3 cells stably expressing AR) cells respectively. Data presented is average ± S.E.M of three independent experiments conducted in triplicate. *p < 0.05.

These results prompted us to examine if Sp1/p65 can mediate androgen regulation of c-FLIP in the absence of AR using AR-negative DU145 cells. Surprisingly, c-FLIP promoter activity was significantly decreased in response to 5α-DHT in these cells (lane 3; Fig. 3A). Consistent with data presented in Fig. 1B, 2-ME_2_ further reduced the observed basal and 5α-DHT-inhibited c-FLIP promoter activity (lanes 2 and 4; Fig. 3A). This unexpected finding prompted us to test if the detected 5α-DHT-mediated effects are related to its metabolism. Therefore, we conducted these experiments using non-metabolizable androgen R1881. Interestingly, R1881 had no significant effect on c-FLIP activation, albeit 2-ME_2_ decreased (lanes 5 and 6; Fig. 3A). Next we investigated if the observed 5α-DHT-mediated decrease in c-FLIP activation is related to reduced Sp1 enrichment at the promoter. ChIP analysis showed significant enrichment of Sp3 and non-significant increase in Sp1 (relative to control IgG antibody) at the endogenous c-FLIP promoter in response to 5α-DHT but not R1881 treatment (lanes 2&7 and 4&9; Fig. 3B). Remarkably, 2-ME_2_-treatment showed 10-fold higher enrichment of Sp3 (relative to control IgG antibody) to the endogenous c-FLIP promoter following 5α-DHT-stimulation (lane 8; Fig. 3B). These results suggest that 5α-DHT metabolic products could be involved in the observed 5α-DHT-induced downregulation of c-FLIP.

Aldo-keto reductase, AKR1C1 predominantly metabolizes 5α-DHT into 3β-Adiol, a physiological ligand for ERβ [21]. We examined alterations in the expression of AKR1C1 and ERβ in DU145 cells and found that 5α-DHT and 2-ME_2_ significantly up-regulated mRNA expression of AKR1C1 and ERβ (Fig. 3C). These correlative results led us to hypothesize that metabolic inactivation of 5α-DHT by AKR1C1 could produce 3β-Adiol that in turn reduces c-FLIP transcriptional activation via ERβ activation. To directly test this, we examined c-FLIP transactivation by ectopically expressing ERβ or pharmacologically by treating cells with 3β-Adiol. Ectopic expression of ERβ (left panel) or treatment with 3β-Adiol (right panel) decreased c-FLIP promoter activity significantly (Fig. 3D). Similar results were also obtained with PC-3 cells (Fig. 3E). In contrast, 3β-Adiol but not ERβ decreased c-FLIP promoter activity in LNCaP cells (Fig. 3E bottom panel). Given the differential status of AR between LNCaP and PC3 & DU145 cells, we reasoned that the presence of AR could be a contributing factor for the observed differential regulation of c-FLIP by ERβ. To address this, we performed similar experiments using PC-3 cells stably overexpressing AR (PC-3 AR). Intriguingly, we did not observe ERβ-mediated down regulation of c-FLIP promoter activity in these cells (Fig. 3F). These data suggest that metabolic inactivation of 5α-DHT by AKR1C1 could produce 3β-Adiol, which in turn reduces c-FLIP transcriptional activation possibly via ERβ activation. Furthermore, AR inhibits ERβ-mediated inhibition of c-FLIP activation. Given the lack of consensus estrogen response element in the c-FLIP promoter, how ERβ suppresses c-FLIP is unclear.

### ERβ competes with Sp1 and Sp3 for binding to c-FLIP promoter

**Figure 4:**
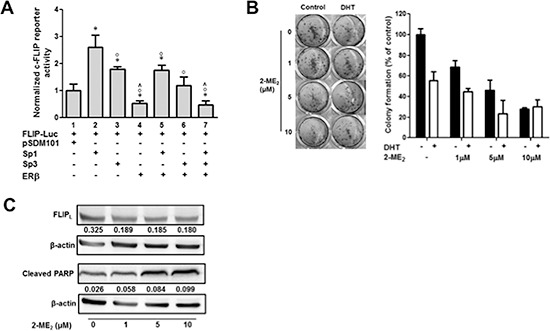
Non-genomic regulation of c-FLIP by ERβ **A.** ERβ suppresses Sp1 and Sp3-mediated transactivation of c-FLIP in DU145 cells. Transient expression was conducted as described in materials and method using pGL3-FLIP (–121/+242)-Luc plasmid in the presence and absence of expression plasmids for Sp1, Sp3 or ERβ. Where indicated cells were co-transfected with expression plasmids for Sp1, Sp3 or ERβ or all three (0.5 μg/well of each) and total amount of DNA was maintained constant using pcDNA3.1. (*p < 0.05; *compared to pSDM101 control, °compared to Sp1, and ^compared to Sp3). **B.** Colony forming ability of DU145 cells stimulated with 5α-DHT. DU145 cells were seeded in 24-well plates at a density of 100 cells per well in triplicate in 1 mL of growth media and stained with crystal violet after treating with 5α-DHT (1 nM) for 1 h and 2-ME_2_ for 24 h (Left), and colonies were counted (Right). Error bars indicate ± S.D. (*n* = 3). **C.** 2-ME_2_ induces apoptosis in DU145 cells. Whole cell extracts prepared from cells treated with increasing concentration of 2-ME_2_ (1, 5, and 10 μM) were used in immunoblotting with antibodies for cleaved PARP (48 h) and FLIP_L_ (12 h). *β*-actin was used as a loading control. Numbers indicate ratio of c-FLIP or c-PARP with respect to *β*-actin loading control.

Previous studies have shown that estrogen receptors can regulate gene expression non-genomically through interactions with other transcription factors such as Sp1 [22, 23]. We tested this hypothesis using co-transfection assays in DU145 cells. Consistent with data presented in Figs. 1E and 3D; ectopic expression of Sp1 trans-activated while Sp3 trans-repressed Sp1-mediated activation (lanes 2 and 3; Fig. 4A). On the other hand, ectopic ERβ expression not only suppressed constitutive & Sp1-mediated activation but also Sp3-mediated inhibition c-FLIP activation (lanes 4–7; Fig. 4A). Furthermore, ectopic ERβ expression further suppressed Sp3-mediated inhibition of Sp1 transactivation (lane 8; Fig. 4A). Although the biological significance of these observations is unclear, these results imply that ERβ suppresses c-FLIP transcriptional activity possibly by competing with Sp1 and Sp3. Biological relevance of 5α-DHT or 2-ME_2_-mediated suppression of c-FLIP activation was determined by examining the effect of 5α-DHT on cell growth and apoptosis in the presence and absence of 2-ME_2_. Treatment with 5α-DHT significantly decreased colony-forming ability of DU145 cells. Decreased colony forming ability was further reduced in the presence of 2-ME_2_ (Fig. 4B). Immunoblot analysis revealed dose-dependent increase in PARP cleavage and decreased FLIP_L_ (Fig. 4C). These observations suggest that ERβ can inhibit growth of androgen-independent prostate cancer cells through transcriptional downregulation of c-FLIP and activation of apoptosis.

**Figure 5:**
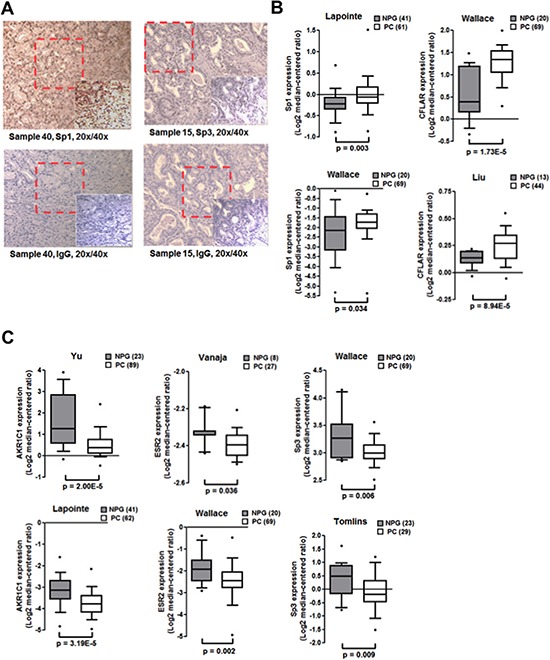
Expression of Sp1, Sp3, c-FLIP, AKR1C1 and ERβ in human prostate tumors **A.** Human prostate tissue array containing 80 tissue cores (duplicated tissues from 40 individual donors) obtained from Cybridi, Inc. was used in immunohistochemical evaluation using antibodies against Sp1, Sp3 (sc-28305) and negative control IgG. Representative picture at different magnifications is shown. **B.** Box plots of *in silico* analysis of expression of Sp1 (left panels) and c-FLIP (right panels) in human prostate cancer and normal prostate tissues. Oncomine microarray data retrieved from two independent cohorts was used. **C.** Box plots of *in silico* analysis of expression of AKR1C1, ERβ and Sp3 expression (left, middle and right panels) in human prostate cancer and normal prostate tissues. (NPG: normal prostate gland, PC: prostate carcinoma).

We analyzed the expression of Sp1 and Sp3 in human prostate tissues by immunohistochemistry (IHC). IHC analysis revealed significantly elevated Sp1 expression (27/35 human prostate tumors; *p* = 0.035) with no detectable expression in the normal tissue (Fig. 5A and Table 1). However, differences in Sp3 expression did not reach statistical significance (Table 1). We also compared the expression of AKR1C1, c-FLIP, Sp1, Sp3 and ERβ in human prostate tumors and normal tissue from oncomine, cancer-profiling database. *In silico* analysis of these data from two different cohorts revealed significantly upregulated expression of Sp1 and c-FLIP in human prostate tumors compared to normal tissue (Fig. 5B). On the other hand, expression of AKR1C1, ERβ and Sp3 was down regulated (Fig. 5C).

**Table 1:** Quantitative analysis of immunohistochemical evaluation of Sp1 and Sp3 Immunohistochemical staining was performed essentially as described in methods. The staining results were scored blindly and semi quantitatively based on staining intensities and proportion of positive staining tumor cells. Briefly, the proportion of positive tumor cells was scored as follows: 0, no stained cells; 1, 1–50%; 2, 50–100% positive staining. The intensity score represents the average staining intensity of tumor cells: +, mild; ++, weak; +++, strong intensity. The statistical significance was calculated using Pearson Chi-Square test.

		Negative	Positive	Total	Pearson Chi-Square
Normal	Sp1	3	0	3	Value 7.995 *p* = 0.035
Carcinoma		8	27	35	
Total		11	27	38	
Normal	Sp3	0	0	0	Value 1.327 *p* = 0.249
Carcinoma		23	11	34	
Total		23	11	34	

## DISCUSSION

Recent evidence shows the importance of androgen metabolism in the progression of prostate cancer to castrate-resistant state. Here, we show for the first time that AKR1C1 can contribute to CRPCA by inhibiting apoptosis through ERβ-mediated transcriptional suppression of c-FLIP. The tumor suppressive role for ERβ has been reported using *in vitro* cell culture and *in vivo* animal models [6–10]. It has also been reported that human prostate cancer cells express both ERα and β and ERβ knockout mice develop HGPIN lesions [9]. Furthermore, *in silico* analysis revealed significantly decreased expression of both AKR1C1 and ERβ in human prostate tumors compared to normal tissue. Therefore, our data demonstrating tumor suppressor role for ERβ is in agreement with these published findings.

Additionally, we provide evidence that ERβ inhibits apoptosis non-genomically by competing with Sp1 and Sp3 to transcriptionally downregulate c-FLIP. Given the lack of understanding how ERβ activation inhibits apoptosis to drive prostate pathogenesis, our observations are novel. Estrogen receptors (ERs) regulate gene expression either classically by binding to estrogen response element (ERE) in the promoter regions or non-classically in association with other transcription factors including AP-1, Sp1 and NFκB [22]. Genome-wide global profiling of ERβ occupancy studies indicate that ERE is not the major binding site for ERβ, but it can bind to sites including Sp1 [23]. Data presented in this manuscript provide evidence that 2-ME_2_ (i) inhibits Sp1 transactivation of c-FLIP; (ii) activates ERβ in a dose-dependent manner and (iii) that ectopic expression of ERβ or treatment with its ligand 3β-Adiol suppresses c-FLIP promoter activity despite lack of consensus ERE. The observed downregulation of c-FLIP is associated with induction of apoptosis in prostate cancer cells. Based on these observations, we speculate that ERβ downregulates c-FLIP either directly by binding to Sp1 sites on the c-FLIP promoter or by modulating the ratio of Sp1/Sp3 through competitive DNA binding. Alternatively, involvement of other ERβ coregulators cannot be ruled out. Additionally, it is also possible that 2-ME_2_-treatment can activate epigenetically silenced ERβ through demethylation. It has been reported that due to methylation of the ERβ promoter, relative ERβ expression is undetectable in LNCaP and very low in DU145 cells compared to PC-3 cells [26]. Since our results show that treatment with 2-ME_2_ resulted in dose-dependent increased ERβ expression in DU145 cells they signify the possibility that 2-ME_2_-treatment can activate epigenetically silenced ERβ through demethylation, which will be a focus of our future studies. Taken together, these observations for the first time suggest that loss of ERβ can contribute to aggressive advanced prostate cancer through transcriptional regulation of c-FLIP.

Sp1 and NFκB are transcription factors that regulate expression of genes involved in various cellular processes of oncogenesis including differentiation, apoptosis, cell migration, and cell cycle progression [27, 28]. Moreover, published studies show correlation between elevated levels of NFκB in castrate-resistant prostate tumors and disease progression [29]. However, to the best of our knowledge no studies have examined the expression of Sp1 and Sp3 in human prostate tumors. The current study for the first time demonstrates elevated expression of Sp1 in human prostate tumors compared to normal tissue. These observations are consistent with published studies in other tumor types [30–32]. For example, in comparison to normal tissues or cells Sp1 levels are higher in breast, thyroid, hepatocellular, pancreatic, colorectal, gastric and lung cancer. In addition, abnormal Sp1 levels are highly correlated with stage and poor prognosis of cancer [31].

Our data also shows potential combinatorial effects of Sp1 with AR, Sp3 and p65 in the regulation of c-FLIP transcription. Treatment with pro-apoptotic agent 2-ME_2_ also blocked the observed effects. Sp1 and Sp3 both bind GC-rich sequences and regulate gene expression either thorough cooperative interactions or inhibitory interactions whereby Sp3 can decrease Sp1 transactivation. Furthermore, published evidence demonstrates that Sp1 can also regulate gene expression in association with ligand activated and orphan nuclear receptors including AR, ER, progesterone receptor (PR) and retinoic acid receptor (RAR). Sp1 and/or Sp3 also cooperatively activate gene expression through interaction with other transcription factors including E2F, SMADs, NFκB, GATA and c-Jun [28]. Previous studies have demonstrated association between Sp1 and AR. For example, it has been shown that despite lack of consensus ARE, AR can transactivate p21 by binding to Sp1 sequence elements in association with Sp1 in response to androgens [33]. In addition, Sp1 stimulates AR target gene PSA through its transcriptional activation ability. Inhibition or knocking down of Sp1 to normal cellular level has been reported to decrease tumor formation, growth and metastasis [34–37]. For example, chemopreventive agents including Betulinic acid and Curcumin reduce the expression of Sp1 and Sp3 with consequent reduced expression of their target genes including EGFR, Cyclin D1, VEGF and SREB2. Mithramycin A and tolfenamic acid together reduce Sp1 levels in pancreatic cancer cells. Thus, interactions between Sp1 and other proteins can differentially affect Sp1-dependent transactivation depending on the promoter context [27, 28]. In agreement with these published findings, our work demonstrates that 2-ME_2_-mediated restoration of ERβ prevents Sp1 or p65 recruitment or combination of both Sp1 and p65 to c-FLIP promoter with consequent induction of apoptosis. In future studies, we will determine whether binding of Sp1 facilitates recruitment of NFκB or vice versa.

**Figure 6:**
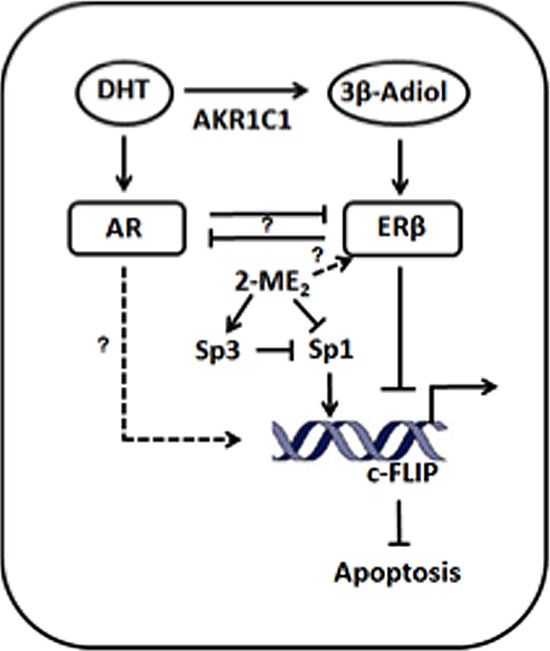
Hypothetical model Hypothetical model of ERβ-mediated transcriptional inhibition of c-FLIP. AKRC1 mediated inactivation of 5α-DHT generates 3-β-Adiol, a potent ligand for ERβ. Activation of ERβ by 3-β-Adiol down regulates c-FLIP transcriptionally possibly by regulating the binding of Sp and NFκB factors to the c-FLIP promoter. Furthermore, 5α-DHT-induced activation of AR could potentially suppress ERβ-mediated transcriptional suppression of c-FLIP activation.

Surprisingly, the current study also observed that treatment with metabolizable androgens such as 5α-DHT but not non-metabolizable R1881 lowers enrichment of Sp1 and increase enrichment of Sp3 to the c-FLIP promoter leading to reduced transcriptional activation and inhibition of colony formation in DU145 cells. How might treatment with 5α-DHT in AR negative cells contribute to growth suppression? AKR family members including AKR1C1, C2 and C3 function as 3-, 17- and 20-ketoseteroid reductases respectively to form 3-α/β, 17-β and 20-α-hydroxyl metabolites [21, 38]. For example, inactivation of 5α-DHT by AKR1C1 results in the formation of 3β-Adiol, a potent ERβ ligand [21]. Interestingly, the intraprostatic levels of 3β-Adiol, was reported to be 100-fold higher than estradiol [21]. Data presented here shows (i) expression of AKR1C1 decreases in human prostate tumors, (ii) 5α-DHT enhances AKR1C1 expression; (iii) both 3β-Adiol and ERβ suppress c-FLIP activation in DU145 and PC-3 cells but not in LNCaP cells. It is noteworthy to mention that PC-3 cells are more sensitive to 3β-Adiol, which could be related to relatively higher expression of ERβ as it is unmethylated. These observations are consistent with the hypothesis that variations in the intraprostatic levels of 3β-Adiol can potentially dictate the outcome of AR/ER signaling and possibly the disparate increased risk of CRPCA by preventing apoptosis via c-FLIP. Hypothetical model is shown in Fig. 6. In future studies, we will determine the crosstalk between ERβ and androgen metabolism and how other nuclear receptors including ERα impact the biological outcome.

Prostate cancer is the second leading cause of cancer related deaths in men. Localized cancer can be treated effectively either by surgery or radiation [39]. The first line therapy, ADT though initially effective, progresses to castrate-resistant disease in a majority of patients [40]. There are no effective curative approaches for treatment of castrate-resistant disease. It is well accepted that AR is activated in about 80% of castrate-resistant tumors [41]. Several mechanisms including AR amplification/overexpression, alteration in cofactors or AR variants and crosstalk with signal transduction pathways plays a critical role in the reactivation of AR [42, 43]. In addition, emerging evidence demonstrates survival benefit and tumor growth inhibition in patients by suppression of adrenal androgen production by abiraterone acetate suggests a pivotal role for intratumoral androgen metabolism [44]. Similarly, enzalutamide, which provides survival benefit directly, inhibits AR activation [45]. Although these data provide convincing evidence that androgen metabolism and AR are critical targets for CRPC progression, resistance to these agents is a major challenge for management of CRPC. This is substantiated by recent data demonstrating reactivation of glucocorticoid receptor (GR) as a prevalent mechanism of acquired resistance to enzalutamide [46]. Data presented in this manuscript provided evidence for targeting c-FLIP either by increasing the levels of AKR1C1 or activating ERβ could have therapeutic potential. Do changes in AKR1C1 lead to increase in c-FLIP expression during CRPCA progression? How do interactions among transcription factors Sp1/Sp3/NFκB/AR involved in upregulation of c-FLIP contribute to CRPCA? Taken together, these observations clearly indicate a conjectural role for AKR1C1/ERβ/c-FLIP in prostate pathogenesis that warrants additional investigations to elucidate the regulation of c-FLIP with respect to androgen metabolism and progression to CRPC. Furthermore, despite its ability to exert anti-neoplastic activity in variety of tumor types, 2-ME_2_ was found to be clinically non-viable [47]. The data presented in this manuscript justifies investigations combining 2-ME_2_ with ERβ agonists or 3β-Adiol for effective management of CRPC.

## MATERIALS AND METHODS

### Cell lines and reagents

AR-positive androgen dependent (LNCaP), AR-negative androgen independent (PC-3 and DU145) human prostate cancer cells were purchased from American Type Culture Collection (Manassas, VA); and AR-positive androgen independent (C4-2B) were obtained from Dr. Thambi Dorai (Department of Biochemistry and Molecular Biology, New York Medical College, NY). All cells were grown essentially as described previously [18]. Logarithmically growing LNCaP, PC-3 and C4-2B (3 μM) and DU145 (5 μM) cells were treated with 2-methoxyestradiol {2-ME_2_; obtained from Sigma-Aldrich (St. Louis, MO)}.

### Western blot analysis and quantitative real time PCR

Western blot and qRT-PCR analysis was conducted as described previously [18]. Primary antibodies used include FLIP_L_ (generated in-house), Cleaved PARP (9541S; Cell signaling, Danvers, MA), and *β*-actin (A5316; Sigma-Aldrich, St. Louis, MO). Bound antibody was visualized using ECL kit (Thermo Fisher Scientific, Waltham, MA). All the blots were stripped and reprobed with *β*-actin to ensure equal loading of protein. Images were captured and analyzed using Gene snap software (Syngene, Frederick, MD), and quantification was carried out using Gene tools software (Syngene, Frederick, MD).

Total cellular RNA isolated using Trizol reagent (Invitrogen, Grand Island, NY) according to the manufacturer's instructions. Target genes were amplified and expression was measured using 7300 Applied Biosystems with SYBR Green dye. The primers used were as follows: *β*-actin, forward 5′-GGCACCCAGCACAATGAAGATCA-A-3′ and reverse 5′-TAGAAGC-ATTTGCGGTGGACGATG-3′; AKR1C1, forward 5′-GCTTTAGAGGCCACCAA-ATTGGCA-3′ and reverse 5′-ACTGCCATCTGCAATCTTGCTTCG-3′; ERβ, forward 5′-GGCACCTTTCTCCTT-TAGTG-3′ and reverse 5′-GGTGTGTTCTAGCGATCTTG-3′. PCR reactions were conducted in triplicate, and relative mRNA expression was normalized to *β*-actin. Fold change in experiments was determined relative to solvent control group. Specific amplification of target genes was validated using a dissociation curve.

### Transient transfection

For transfections, human prostate cancer cells were plated in triplicate at a density of 100,000 cells per well in 24-well plates. Cells were transfected with indicated c-FLIP reporter plasmids (0.5 μg) along with *Renilla* luciferase (10 ng) using Lipofectamine 2000 reagent (Invitrogen, Grand Island, NY) according to the manufacturer's recommendations. For co-transfection experiments, 0.5 μg/well of Sp1, Sp3, and/or ERβ was used along with –121/+242 sequence of c-FLIP reporter plasmid. Total amount of DNA was maintained constant using backbone vector pcDNA3.1. Where necessary, cells were treated with or without 2-ME_2_ for 24 h and luciferase activity was determined using Dual Luciferase Reporter Assay system (Promega, Madison, WI).

### Electrophoretic mobility shift assay (EMSA) and chromatin immunoprecipitation (ChIP)

EMSA and ChIP were essentially conducted as described previously [13, 19]. For EMSA, Nuclear extracts prepared from LNCaP, C4-2B, and PC-3 cells treated with 2-ME_2_ for 24h were used in EMSA experiments using ^32^P-labeled c-FLIP promoter sequence oligonucleotide (+64/+89) containing overlapping binding sites for Sp1, Sp3, p65 and AR. For competition experiments, the radiolabeled probe was mixed with 100-fold molar excess of unlabeled double-stranded synthetic c-FLIP oligonucleotide for 5 min prior to the addition of nuclear extracts. For super-shift experiments, nuclear extracts were pre-incubated with Sp1, Sp3, p65, p50, AR or control IgG antibodies for 30 min on ice prior to use in EMSA. For ChIP, sheared, cross-linked protein-DNA fragments were immunoprecipitated with normal rabbit IgG (sc-2027), anti-Sp1 (sc-59 X) anti-Sp3 (sc-644 X) antibody, or anti-p65 antibody (sc-372 X; obtained from Santa Cruz Biotechnology, Santa Cruz, CA) and immune complexes were absorbed with protein G magnetic beads (Active Motif, Carlsbad, CA). 10% of the input extract was saved as input control for normalization before adding antibody for immunoprecipitation. Cross-linking was then reversed, and immunoprecipitated DNA was amplified by PCR or quantitative PCR. PCR products were resolved on 3% agarose gel. Densitometry was used to quantify the PCR products and the results were normalized to respective input values. For qPCR, triplicate PCR reactions were performed for each sample and the data are presented as the average ± S.E.M. and the results were normalized to respective input values. Fold enrichment was calculated as 100*2^−(*Ct[Target]−Ct[Input]*)^.

### Clonogenic cell survival assay

For colony formation, DU145 cells seeded in 24-well plates at a density of 100 cells per well in 1 ml of media were treated with 2-ME_2_ for 24 h in the presence or absence of DHT (1 nM towards final 1 h of 24 h treatment). Following this treatment, cells were washed with PBS and media was replaced with fresh media with no treatment. Following one week, cells were fixed with ice-cold 100% methanol and stained with 0.5% crystal violet in 20% methanol, and groups in excess of 15 cells were counted as colonies.

### Immunohistochemical evaluation of Sp1 and Sp3 in human prostate tumors

Human prostate tissue array containing 80 tissue cores (duplicated tissues from 40 individual donors) obtained from Cybridi, Inc. was used in immunohistochemical evaluation. Antibodies for Sp1 (sc-59) and Sp3 (sc-28305) and negative control IgG antibody obtained from (Santa Cruz Biotechnology, Santa Cruz, CA) and (Southern Biotechnology, Cat# 0111-01) respectively were used in immunohistochemical evaluation. Immunohistochemical staining and evaluation was performed by Cybridi, Inc. The staining results were scored blinded and semi quantitatively based on staining intensities and proportion of positive staining tumor cells. Briefly, the proportion of positive tumor cells was scored as follows: 0, no stained cells; 1, 1–50%; 2, 50–100% positive staining. The intensity score represents the average staining intensity of tumor cells: +, mild; ++, weak; +++, strong intensity. The statistical significance was calculated using Pearson Chi-Square test.

### Oncomine data

Sp1, Sp3, c-FLIP, ERβ, and AKR1C1 expression in normal prostate gland and prostate carcinoma were obtained from two independent studies for each gene expression in the Oncomine database. Primary sources are from different group's microarray data mentioned in the graph (http://www.oncomine.org). Data sets are log transformed and illustrated as median centered box plots between the differences of mRNA transcription within cohorts. Statistical significance was determined by a two-tailed Mann-Whitney test. Detailed information of the standardized normalization and statistical calculations are indicated on the Oncomine website.

### Statistical analysis

All numerical results are expressed as mean ± S.D. or S.E.M. derived from 3 independent experiments, unless otherwise stated. Statistical analyses were conducted using Student's *t*-test and statistically significant differences were established as *p* < 0.05. The statistical significance of IHC data was calculated using Pearson Chi-Square test.

## SUPPLEMENTARY FIGURE


