# Supplementary material for: Activation of AKR1C1/ERβ induces apoptosis by downregulation of c-FLIP in prostate cancer cells: A prospective therapeutic opportunity
Source: Oncotarget. 2015 Mar 24;6(13):11600–13. doi: 10.18632/oncotarget.3417 (PMC4484479; doi:10.18632/oncotarget.3417)
Supplement: Supplementary file 1 [file oncotarget-06-11600-s001.pdf]

SUPPLEMENTARY FIGURE

A

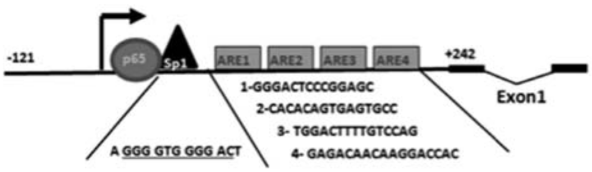

B

S. info.	Transcription factor	Binding site	Length	Application (PCR)	Primer sequences
ChIP primer	Sp1/NFκB	+9F/+157R	180bp	Forward	5'-TAG CAG GAA ACA GCG AGC TT-3'
				Reverse	5'-GCG CTT CTC TCC TAC ACC TC-3'

C

EMSA probe	Transcription factor	Region		Sequence
FLIP	Sp1/NFκB/AR	+64/+89	Upper	5'-CCG GAG CTA GGG GTG GGG ACT CGG-3' (AR, Sp1, p65)
			Lower	5'-CCG AGT CCC CAC CCC TAG CTC CGG-3'

D

EMSA probe	Transcription factor	Region		Sequence
ARE-1	Sp1/NFκB	+57/+71	Upper	5'-GGG ACT CCC GGA GCT-3'
			Lower	5'-AGC TCC GGG AGT CCC-3'
ARE-1m		+57/+71	Upper	5'-GAG GTT CCC GAA GTC-3'
			Lower	5'-GAC TTC GGG AAC CTC-3'

Supplementary Figure S1: A. Schematic representation of potential transcription factor binding sites on the c-FLIP promoter (−121/+242) predicted by Genomatrix program. B. Primer sequences used for ChIP. **C. and D.** Oligonucleotide sequence of c-FLIP sequence, and c-FLIP ARE-1 sequence used in EMSA.
